# Comparison of acid exfoliators in carbon nanosheets synthesis from stinging nettle (*Urtica dioica*) for electrochemical applications

**DOI:** 10.1038/s41598-020-74286-4

**Published:** 2020-10-14

**Authors:** Kanokon Nuilek, Winadda Wongwiriyapan, Vichuda Sattayarut, Andrea Simon, Daniel Koncz-Horváth, Tibor Ferenczi, Ferenc Kristály, Peter Baumli

**Affiliations:** 1 Institute of Ceramics and Polymer Engineering, University of Miskolc, Miskolc, 3515 Hungary; 2 Institute of Metallurgy, Metal Forming and Nanotechnology, University of Miskolc, Miskolc, 3515 Hungary; 3 College of Nanotechnology, King Mongkut’s Institute of Technology Ladkrabang, Chalongkrung Rd., Ladkrabang, Bangkok, 10520 Thailand; 4 Institute of Metallurgy, University of Miskolc, Miskolc, 3515 Hungary; 5 Institute of Mineralogy and Geology, University of Miskolc, Miskolc, 3515 Hungary

**Keywords:** Energy science and technology, Materials science

## Abstract

Carbon nanosheets (CNs) were successfully synthesized from nettle stem (NS) which is an inexpensive material with a high carbon content that is abundantly available in nature. CNs were produced using chemical (potassium hydroxide activation and acid exfoliation) and thermal treatments. Sulfuric (H_2_SO_4_), phosphoric (H_3_PO_4_) and nitric (HNO_3_) acid solutions were used for exfoliation. CNs exfoliated by H_3_PO_4_ have higher specific surface area (789 m^2^ g^−1^) compared to CNs exfoliated by H_2_SO_4_ (705 m^2^ g^−1^) and HNO_3_ (106 m^2^ g^−1^). In this work, NSCNs were found to be a potential candidate for electrode material in electrochemical capacitors. The maximum specific capacitance of the NSCNs exfoliated by H_3_PO_4_ is found to be 27.3 F g^−1^ at a current density of 0.05 A g^−1^, while the specific capacitance of NSCNs exfoliated by H_2_SO_4_ and HNO_3_ is 9.34 F g^−1^ and 1.71 F g^−1^, respectively. Energy density (0.06–0.95 Wh kg^−1^) and power density (20.9–26.7 W kg^−1^) of NSCNs are confirmed to be supercapacitor materials and can be applied in energy storage devices.

## Introduction

2D carbon nanostructures – namely graphene and carbon nanosheets – are increasingly being researched as candidates for energy storage devices such as batteries or capacitors^1–3^. Carbon nanotubes, nanofibers and nanosheets^4–7^ have been well documented. Glass-like carbon is a vital type of the carbon family, typically referred to as glassy carbon or vitreous carbon. Glassy carbon, described as amorphous carbon, is non-graphitizing and contains mostly sp^2^ sites. The properties of carbon are a combination of those of glass and ceramic materials, characterized by low density, high thermal and chemical resistance^8,9^. The preparation of carbon nanostructures is carried out by chemical and thermal treatments. Potassium hydroxide (KOH) activation is a well-known method to generate a pore network in carbons and to expand the carbon layers. Chemical acids are used as intercalating agents to obtain exfoliated graphite by different processes^10–13^. This activated carbon can be obtained from various agricultural wastes^14,15^.

The preparation of nanostructured materials from waste materials has drawn tremendous interest in recent years^16,17^. Many researchers have demonstrated that carbon materials for use as adsorbent or electronic materials can be synthesized by potassium hydroxide from low-cost waste materials such as rice husk^18^, Acai stone^19^, corncob^20^, pine cone flower^21^, banana peel^22^, water hyacinth^23^, waste coffee grounds^24^, pineapple leaf fibre^25^, bamboo^26^, wood sawdust^27^, peanut shell^28–30^, jute^31^, gulfweed^32^ and pomelo peel^33^. Nettle (*Urtica dioica*) is a plant widely distributed in many areas of Asia, Europe, America and Africa^34^. Nettle is considered as waste material mostly used for agricultural purposes or discarded as garbage. Natural structures of nettle consist of cellulose, hemicellulose and lignin^35^, which can be an important precursor in the preparation of highly ordered carbons and contribute to the porosity of biochar yield^36,37^. Moreover, both nettle stems and leaves can be used to produce carbon. Hierarchical porous carbon derived from nettle leaves has been studied for used in advanced supercapacitors and lithium-ion batteries^38^.

One promising area of application for activated carbon is in energy storage and delivery. Ultracapacitors, also called electric double-layer electrodes, are the new range of supercapacitors and are highly efficient in energy storage and delivery characteristics compared to batteries. They are able to deliver high rates of energy involving a mechanism of simple charge separation at the interface between electrode and electrolyte^39,40^. As follows from Ragone plots of the power density against energy density, the efficiency of a supercapacitor depends on the electrode material. Hence, the quest for novel materials for electrodes^41^ involves activated carbon materials owing to their high specific surface area and charge storage^42^. No previous studies have reported on using nettle as a raw material for producing carbon nanosheets by chemical activation and exfoliation process. The activated carbon produced in this way can be used as adsorbent or electrode materials for supercapacitors.

The goal of this work is to systematically investigate the mechanism of different acid activations during the exfoliation process and their effects on the properties of synthesized carbon nanosheets prepared from waste nettle stem. Phosphoric, sulfuric and nitric acid are used in the experiments to examine the influence of triprotic, diprotic and monoprotic acids on the synthesis of carbon nanosheets.

## Results and discussion

### Organic, chemical composition and microstructure of materials

Nettle is mainly composed of cellulose, lignin and hemicellulose (49.8 wt.%, 11.9 wt.% and 15.3 wt.%, respectively). The results of the chemical composition obtained from EDS are listed in Table 1. Due to its high cellulose and carbon content, nettle stem was used to prepare carbon nanostructure, as its carbon content can be further increased by the activation and carbonization processes^17,43^.

**Table 1:** Chemical composition (EDS) of the dried NS, activated NS, NSCNs exfoliated by HNO_3_, H_2_SO_4_ or H_3_PO_4_.

Sample		Chemical composition (wt.%)		
activation	Exfoliation	C	O	Other
Dried nettle stem	50.80	36.03	13.17	
KOH	-	93.84	4.78	1.38
KOH	HNO_3_	88.52	10.51	0.97
KOH	H_2_SO_4_	88.71	9.38	1.91
KOH	H_3_PO_4_	90.96	7.70	1.34

**Figure 1:**
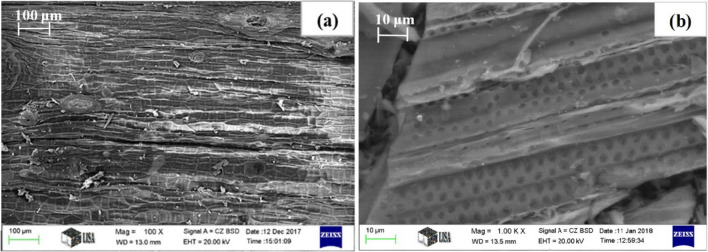
SEM micrographs of (**a**) dried and (**b**) KOH activated nettle stems.

The microstructure of the dried nettle stem (Fig. 1a) reveals a groove and hollow surface, composed of a fibrous structure with many hollow stinging hairs called trichomes. The cell wall in the fibre is inhomogeneous, layered and mainly composed of cellulose, lignin and hemicellulose. After activation, a large number of angular and flake particles and micropores were found on the surface (Fig. 1b). Potassium reacts intensely with the dried nettle stem and pulls apart the layers. Activation increases the carbon content to 93.84 wt.% with a small amount of contaminants.

**Figure 2:**
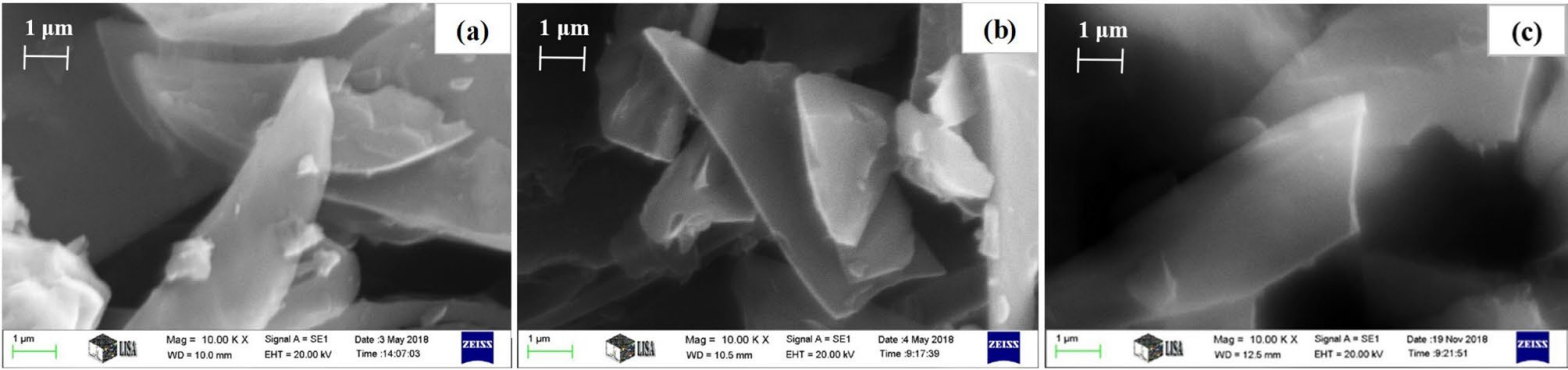
SEM micrographs of the NSCNs exfoliated by (**a**) H_2_SO_4_ (**b**) H_3_PO_4_ and (**c**) HNO_3_.

The exfoliation process was carried out by using one of three different acids: sulfuric, phosphoric and nitric acid. The SEM micrograph of NSCNs exfoliated by H_2_SO_4_ (Fig. 2a) shows a smooth surface and clearly reveals the formation of separated carbon nanosheets with thickness ranging from ~ 42 to 71 nm. EDS results demonstrated that carbon nanosheets mainly contain carbon (88.71 wt.%). In the structure of NSCNs exfoliated by H_3_PO_4_ (Fig. 2b), ultra-thin structures and overlapping carbon nanosheets were identified, with the thickness of carbon nanosheets ranging from 49 to 60 nm. Exfoliation with phosphoric acid yielded the highest carbon content (90.96 wt.%) among the exfoliated specimens. Angular, thin sheets were found in NSCNs exfoliated by HNO_3_, with thicknesses varying from 89 to 95 nm (Fig. 2c), and 88.52 wt.% carbon.

The structure of the produced carbon nanosheets after exfoliating by three different acids are quite similar (Fig. 2a–c); the carbon nanosheets are separated and their surface is clean. The major presence of the element carbon indicates the high purity of these samples. All exfoliating agents (H_2_SO_4_, H_3_PO_4_ and HNO_3_) were suitable to disrupt the layers in the material. During the exfoliation process, acids intercalated into the carbon layers and ruptured interlayer bonds. The exfoliation process increased the specific surface area of the carbonaceous materials (Fig. 5).

**Figure 3:**
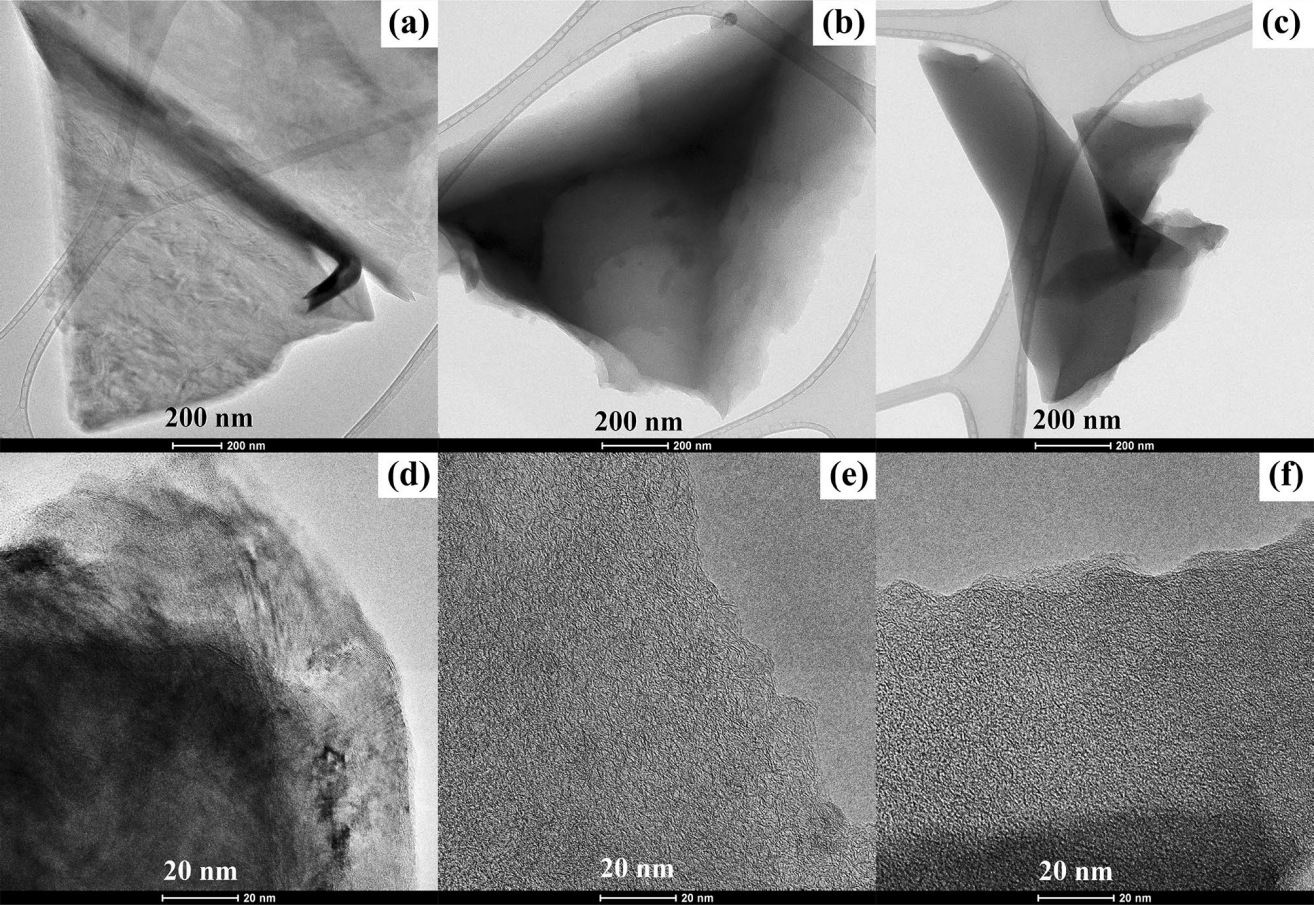
TEM (**a**–**c**) and HRTEM (**d**–**f**) micrographs of the NSCNs exfoliated by (**a**, **d**) H_2_SO_4_. (**b**, **e**) H_3_PO_4_ or (**c**, **f**) HNO_3_.

TEM images (Fig. 3a–c) reveal the amorphous two-dimensional nanosheet porous structure of NSCNs. In the TEM images, the bright and transparent regions are the ultrathin nanosheet and the less transparent areas reveal the overlapped or folding areas of NSCNs, which further imply the ultrathin structure. The structure of the exfoliated NSCNs was explored further with high resolution TEM (HRTEM) imaging (Fig. 3d–f). The layered nanosheet structure is highly porous and gradually becomes thinner toward the edges of the material. NSCNs have a monolayer property, further demonstrating their ultrathin nature^4^. The pore distribution can be seen by the white dots in the grey areas from HRTEM images^3^.

### Structure of carbon nanosheets

**Figure 4:**
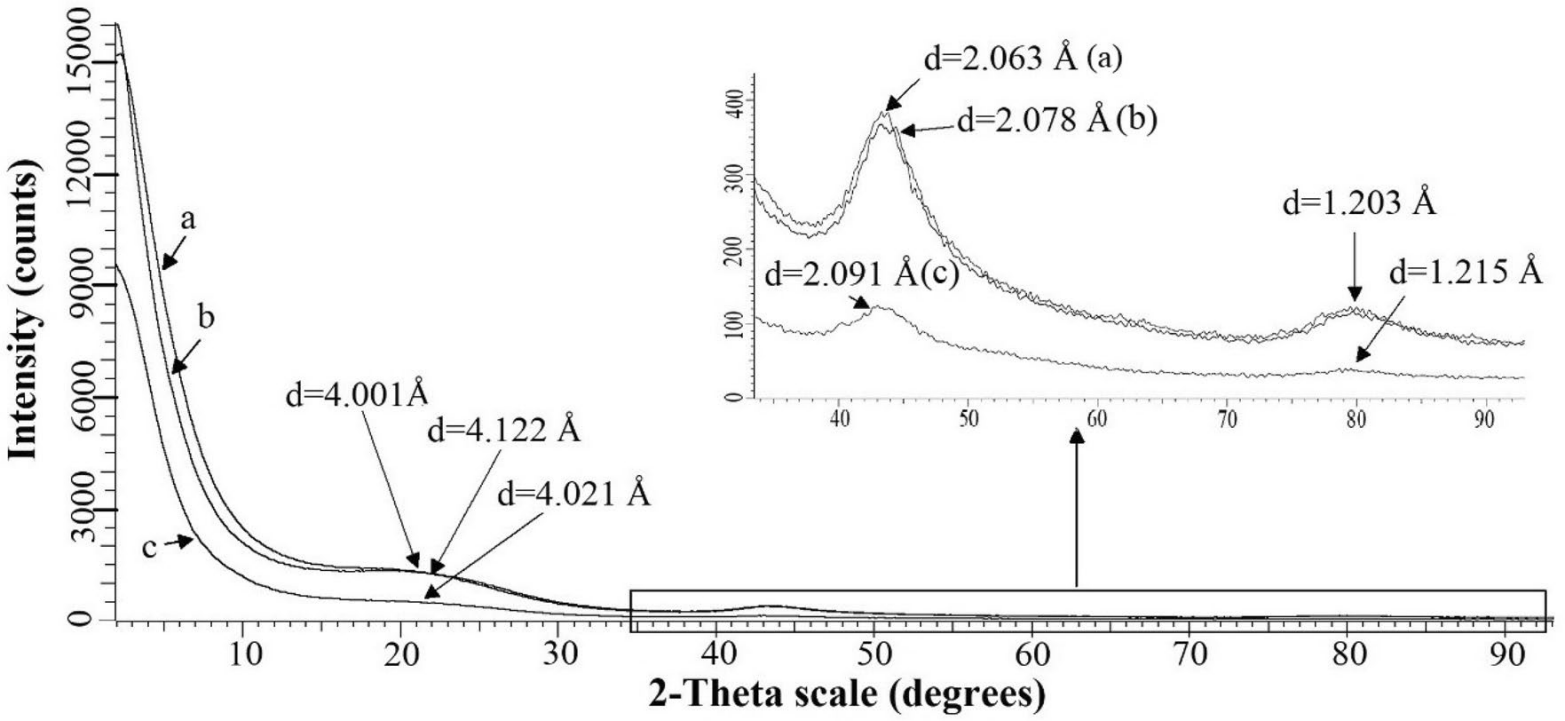
XRD patterns of nettle stem carbon nanosheets exfoliated by (**a**) H_2_SO_4_, (**b**) H_3_PO_4_ or (**c**) HNO_3_.

Figure 4 exhibits X-ray diffractograms of NSCNs. A glassy carbon structure^44–46^ develops in the exfoliated samples. Both the large peaks at 2θ =  ~22° and 44° and the weak and broad diffraction peak occurring in the 2θ range 79°–81° (Table 2) can be attributed to the typical glassy amorphous carbon structure. The XRD patterns of the different samples are quite similar. This suggests that the exfoliating acid does not have a significant effect on the structure of the nettle stem carbon nanosheet. Moreover, the acid and KOH solution do not dissolve the carbon material.

**Table 2:** Peak positions and d values of XRD pattern of NSCNs exfoliated by different acids.

Exfoliating acid	d-value (Å)		
	1st (2θ = ~ 22°)	2nd (2θ = 44°)	3rd (2θ = ~ 80°)
H_2_SO_4_	4.001	2.063	1.203
H_3_PO_4_	4.122	2.078	1.203
HNO_3_	4.021	2.091	1.215

### Surface properties

Physical properties including the specific surface area and the micropore volume of NSCNs were measured using the Brunauer–Emmett–Teller (BET) method based on nitrogen gas adsorption and desorption, as porous materials are also defined in terms of their adsorption properties. The specific surface area and micropore volume of dried nettle stem are found to be 0.17 m^2^ g^−1^ and 0.0001 cm^3^ g^−1^, respectively. The specific surface area of NSCNs exfoliated by H_3_PO_4_, H_2_SO_4_ and HNO_3_ are 789, 705 and 106 m^2^ g^−1^, respectively. Micropore volume of NSCNs exfoliated by H_3_PO_4_, H_2_SO_4_ and HNO_3_ is 0.33, 0.29 and 0.04 cm^3^ g^−1^, respectively. Pore diameters in the exfoliated NSCNs were less than 3 nm. The effect of the phosphoric acid on the surface area of the carbon is stronger than that of the nitric and sulfuric acid by 10.6% and 86.5%, respectively. On the other hand, phosphoric acid has more effect on the change of carbon microporous volume than nitric and sulfuric acid by 9.4% and 87.5%, respectively.

**Figure 5:**
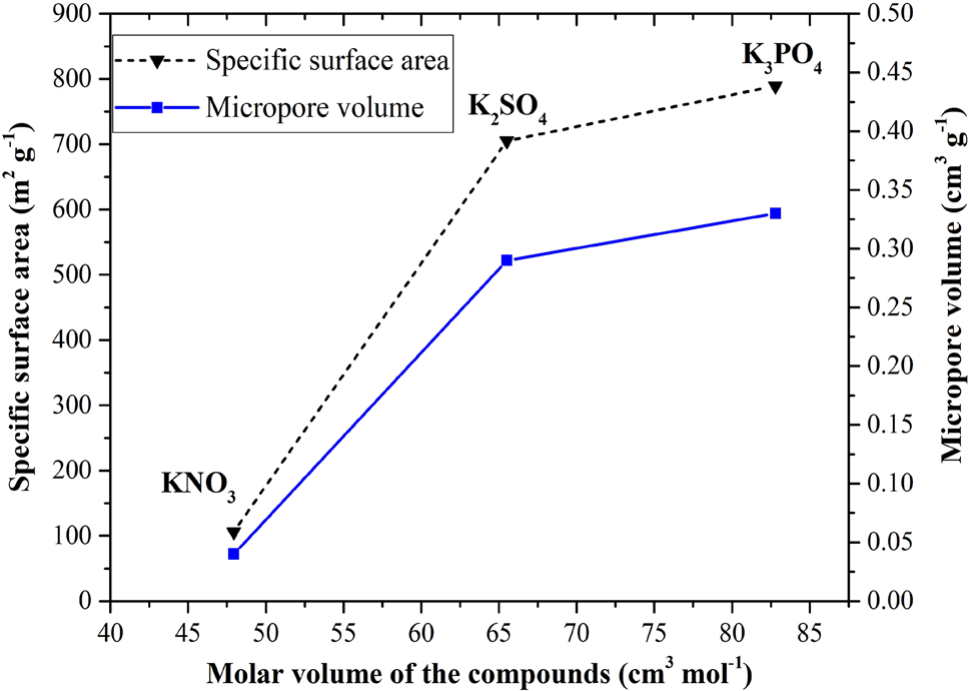
Relationship between molar volume of the compounds versus specific surface area and micropore volume of NSCNs after activation and exfoliation.

During activation, KOH can penetrate the pores of the carbonized material. Interlayered KOH residue can react with the exfoliation acids (H_2_SO_4_, H_3_PO_4_ and HNO_3_), thus some chemical compounds, such as K_2_SO_4_, K_3_PO_4_ and KNO_3_, may form. The molar volume of K_3_PO_4_, K_2_SO_4_ and KNO_3_ is 82.79, 65.51 and 47.94 cm^3^ mol^−1^, respectively. Based on their thermochemical properties (from the database of HSC Chemistry)^47^, all these compounds can form due to negative Δ_f_G. The Δ_f_G of K_3_PO_4_, K_2_SO_4_ and KNO_3_ is − 1876.38, − 1319.67 and − 394.70 kJ mol^−1^, respectively. These compounds cause stress in the pores of the activated carbon. As K_3_PO_4_ has a 26.4 or 72.7% higher molar volume than K_2_SO_4_ or KNO_3_, respectively, it generates the highest specific surface area and micropore volume in KOH activated carbon nanosheets (Fig. 5).

**Figure 6:**
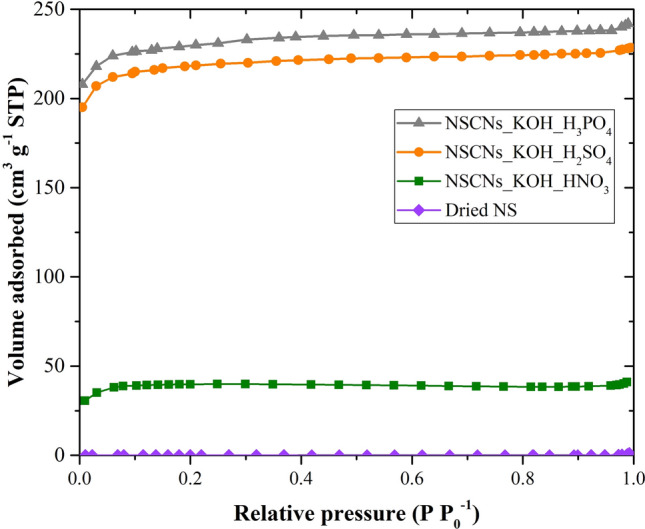
Nitrogen adsorption isotherms of dried NS, NSCNs exfoliated by HNO_3_, H_2_SO_4_ or H_3_PO_4_.

Based on the results of nitrogen adsorption, pore structure can be divided into 6 classes according to the IUPAC classification^48^. The results from nitrogen adsorption isotherms (Fig. 6) show porous structure properties of carbons, which can be changed due to chemical activation and exfoliation. The dried nettle stem shows nonporous and macroporous property with low specific surface area. NSCNs after activation and exfoliation process can be classified as micropore materials with type I isotherm. NSCNs after exfoliating by H_2_SO_4_ and H_3_PO_4_ can be classified as wider micropore materials with type I (a) isotherm. However, NSCNs after exfoliating by HNO_3_ have a quite different pore structure, becoming a type I (b) isotherm, showing wider micropores with a contribution of narrow mesopores, respectively. The qualitative property of carbon nanosheets was confirmed by SEM images and BET. The two investigations assure that carbon nanosheets were formed with a higher specific surface area (which can describe the separation ability of the carbon layer from this process) and small pore diameter.

The adsorption capacity of activated carbons is related to the surface area, pore volume, and pore size distribution. Generally, the adsorption capacity increases as the surface area of activated carbon is increased. The micropore volume of NSCNs after exfoliating by sulfuric acid and phosphoric acid is clearly higher than that of dried nettle stems. The activation and exfoliation process increased the micropore volume of the sample in the same way as the specific surface area was increased. Phosphoric acid affected the surface of NSCNs more than sulfuric acid or nitric acid due to the fact that a polyprotic acid is capable of donating more than one proton. Phosphoric acid is a triprotic acid^49^ having three dissociable protons, and all three protons can be successively lost to yield H_2_PO_4_^−^ followed by HPO_4_^2−^ and finally PO_4_^3−^. A triprotic acid reacts with the materials more intensely than a diprotic (sulfuric acid) or monoprotic (nitric acid). The reactions result in a higher micropore volume (a more porous carbon surface), higher specific surface area and higher specific volume.

**Figure 7:**
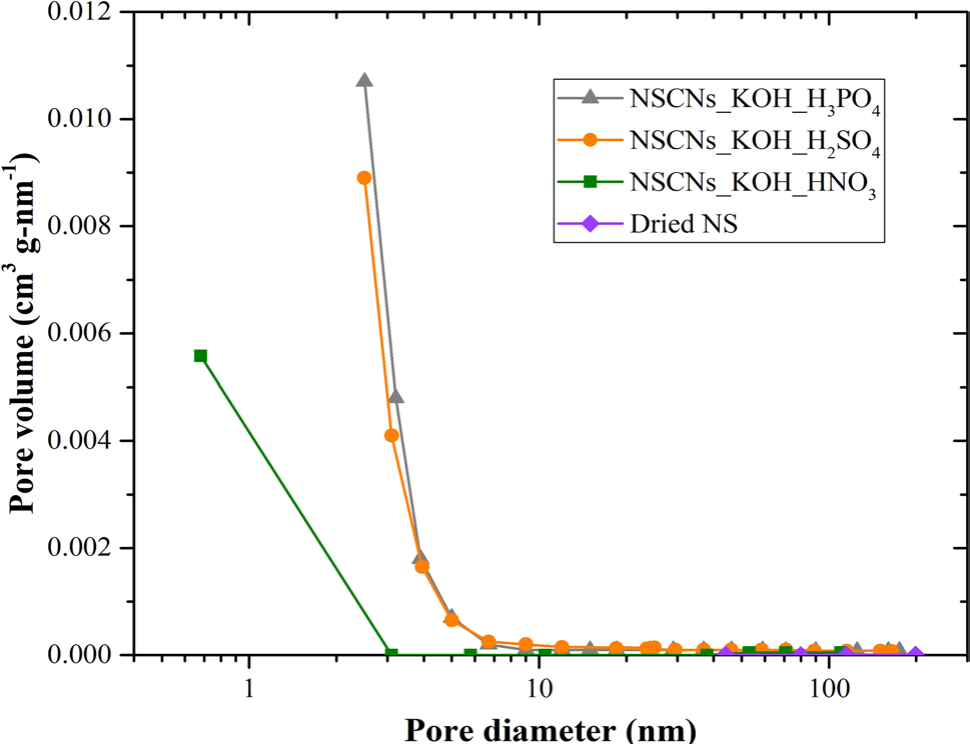
Pore size distribution (BJH adsorption dV/dD) of dried NS, NSCNs exfoliated by HNO_3_, H_2_SO_4_ or H_3_PO_4._

During exfoliation, cations remained in carbonaceous materials after activation reacted with the acids. The pore size distribution (Fig. 7) of NSCNs exfoliated by H_2_SO_4_ or H_3_PO_4_ is relatively uniformed, consisted of wider micropores and mesopores distributed in 2–10 nm while NSCNs exfoliated by HNO_3_ mainly consisted of mesopores and small volume of micropores, and nonporous or macropores for dried nettle stem. The results were in accordance with those of the adsorption isotherms.

The BET measurements confirmed the formation of carbon nanosheets with a high specific surface area (which can describe the separation ability of carbon layer) and small pore diameter and high micropore volume. The highest specific surface area and micropore volume of NSCNs were obtained after activating with KOH and exfoliating by phosphoric acid.

### Electrochemical properties

**Figure 8:**
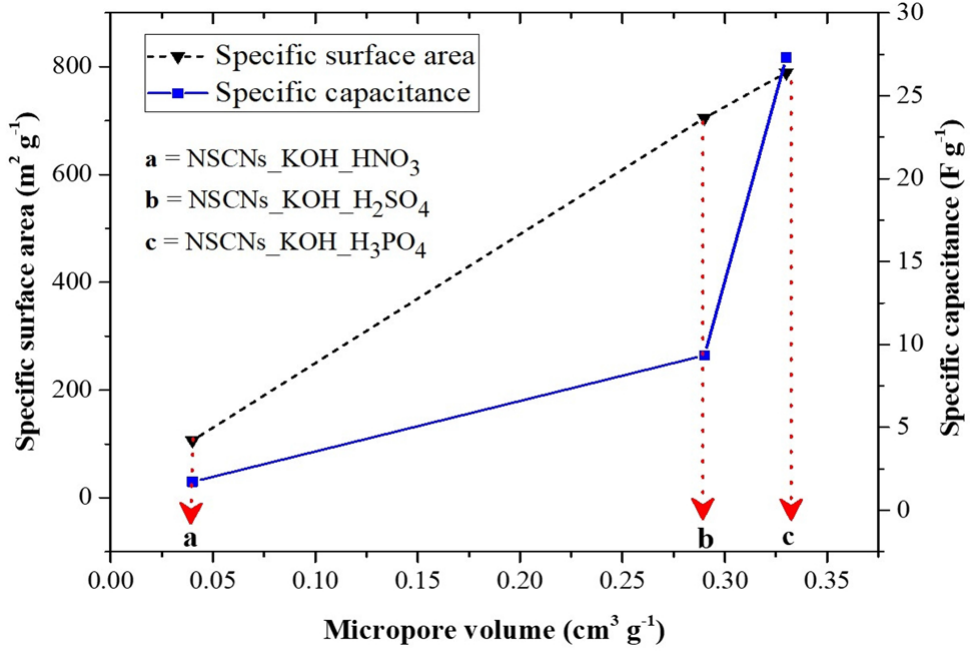
Specific surface area and specific capacitance of samples as a function of micropore volume.

**Figure 9:**
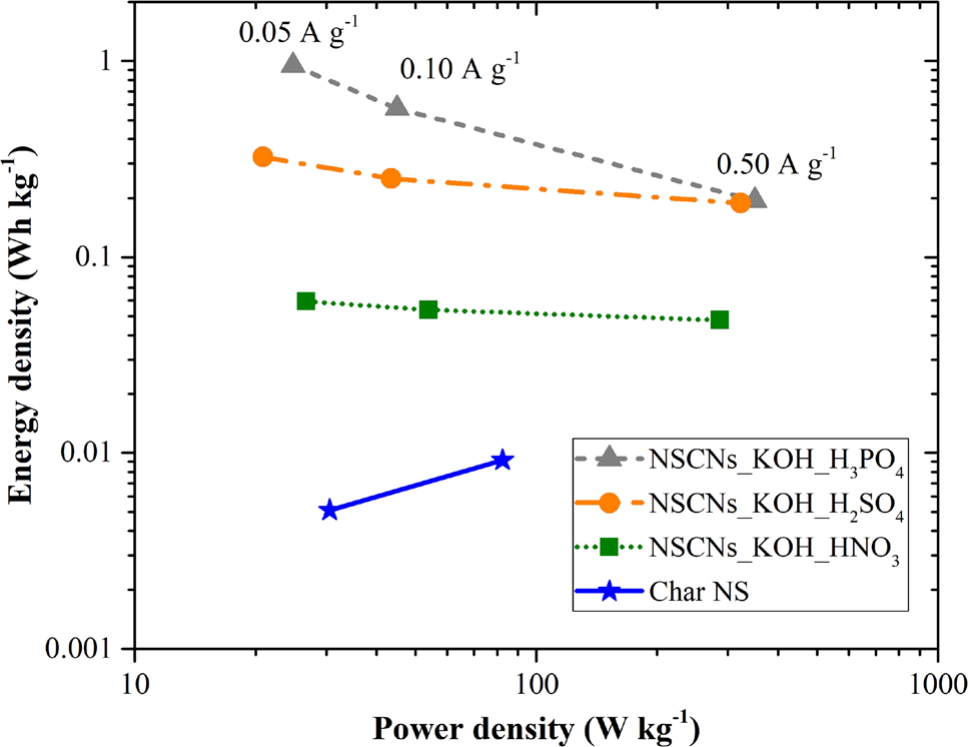
Energy density as a function of the power density of samples.

Figures 8 and 9 present the specific capacitance, energy density and power density characteristic of the electrode. The specific capacitance of NSCNs exfoliated by H_3_PO_4_, H_2_SO_4_, HNO_3_ and that of char NS at a current density of 0.05 A g^−1^ was found to be 27.3, 9.34, 1.71 and 0.15 F g^−1^, respectively. The largest specific capacitance was exhibited by the NSCNs exfoliated with H_3_PO_4_. The nitrogen adsorption isotherm of NSCNs (Fig. 6) showed mostly a micropore structure according to the IUPAC classification. Micropores contain bottlenecks that can decrease ion mobility drastically, thus reducing the power capability of the electrode^50^. Porosity and surface area are directly related to capacitance, as the charge stored on the electrode surface depends on the contact established between electrode and electrolyte^51^.

The specific surface area and specific capacitance of the activated and exfoliated NSCNs increase with micropore volume. The highest specific capacitance (27.3F g^−1^) was found in NSCNs activated with KOH and exfoliated by H_3_PO_4_ (Fig. 8). Figure 9 shows Ragone plots demonstrating the power density and energy density of the supercapacitor electrodes. The NSCNs exfoliated by H_3_PO_4_ could deliver the highest energy density of 0.95 Wh kg^−1^ at a power density of 25 W kg^−1^ (at a current density of 0.05 A g^−1^) and the highest power density of 350 W kg^−1^ at the energy density of 0.19 Wh kg^−1^ (at a current density of 0.50 A g^−1^).

**Figure 10:**
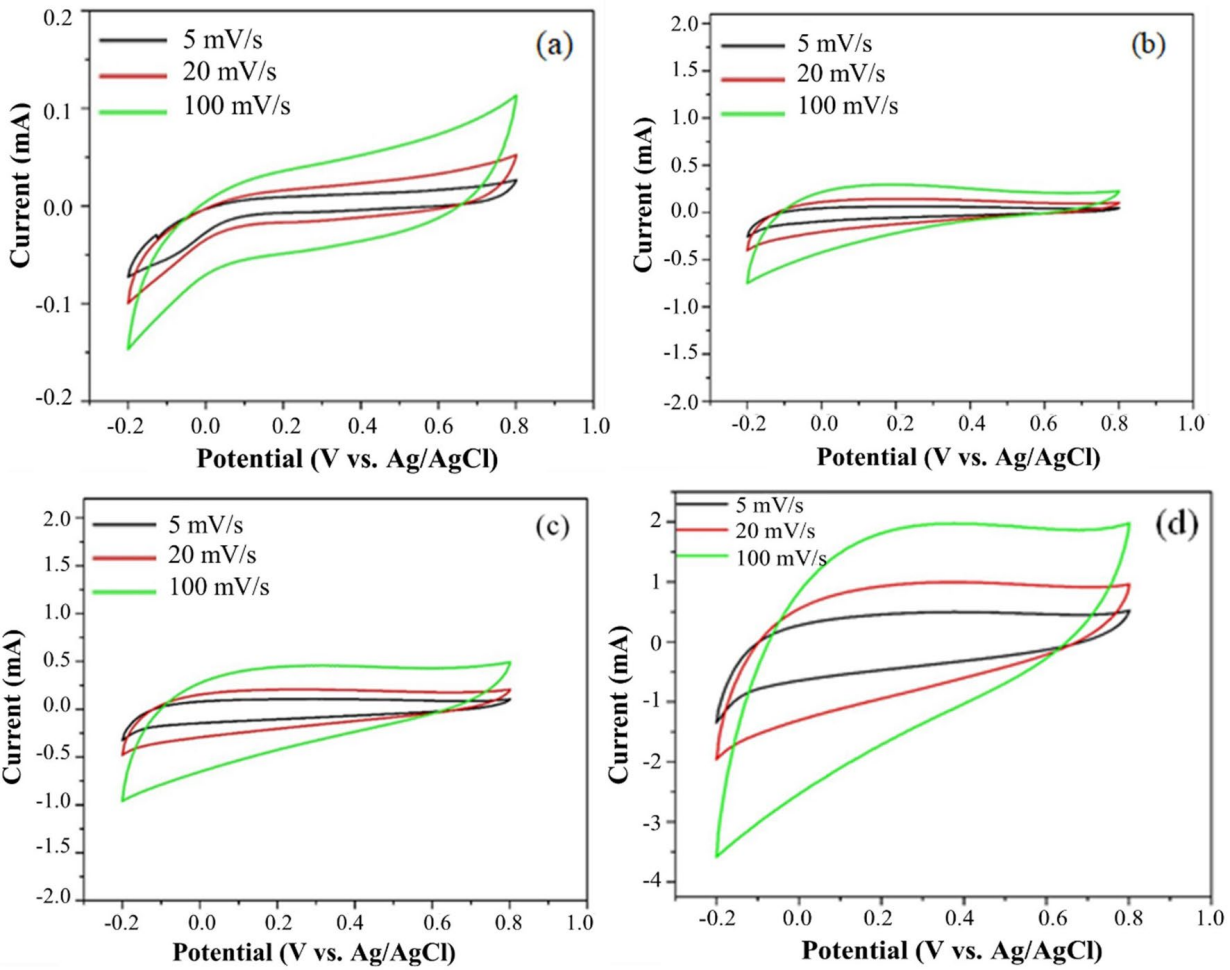
Cyclic voltammetry (CV) of nettle stem carbon nanosheets, (**a**) char NS, (**b**) exfoliated by HNO_3_, (**c**) exfoliated by H_2_SO_4_ and (**d**) exfoliated by H_3_PO_4._

**Figure 11:**
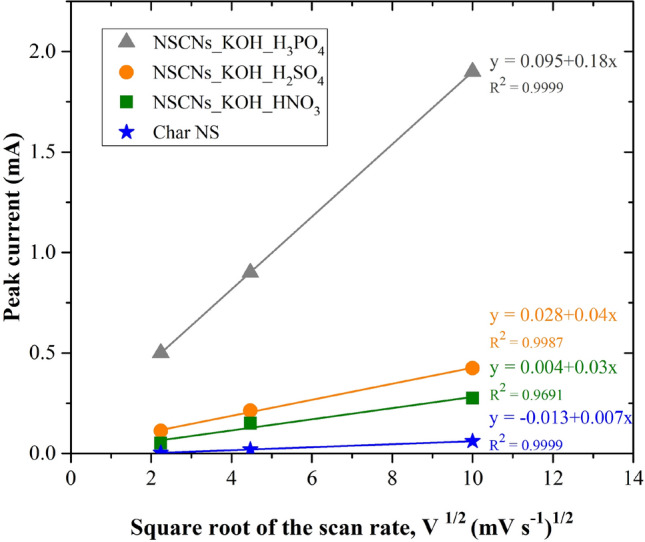
Relationship between peak current as a function of square root of the scan rate of cyclic voltammetry measurement.

Cyclic voltammetry (CV) measurements at 5, 20 and 100 mV s^−1^ in the potential range − 0.2 to 0.8 V were used to calculate the specific capacitance of carbons. CV curves show a quasi-rectangular shape at increased scan rates, indicating that the NSCNs can be used in energy storage applications^52^. The CV shape for NSCNs exfoliated with H_2_SO_4_ and H_3_PO_4_ (Fig. 10c, d) is more rectangular, revealing a better charge propagation compared to char NS (Fig. 10a), while NSCNs exfoliated with HNO_3_ (Fig. 10b) showed a distorted quasi-rectangular shape. The quasi-rectangle shapes maintained even at a high scan rate of 100 mV s^−1^, which is suitable for a typical, stable double layer capacitor that is quick and efficient in charge transfer. They also have excellent capacitive behaviour^53^. Plotting the anodic peak current (inflection point current) against square root of the scan rate shows a linear correlation (see Fig. 11), which indicates that the electrochemical process is limited by the rate of diffusion^54^.

**Figure 12:**
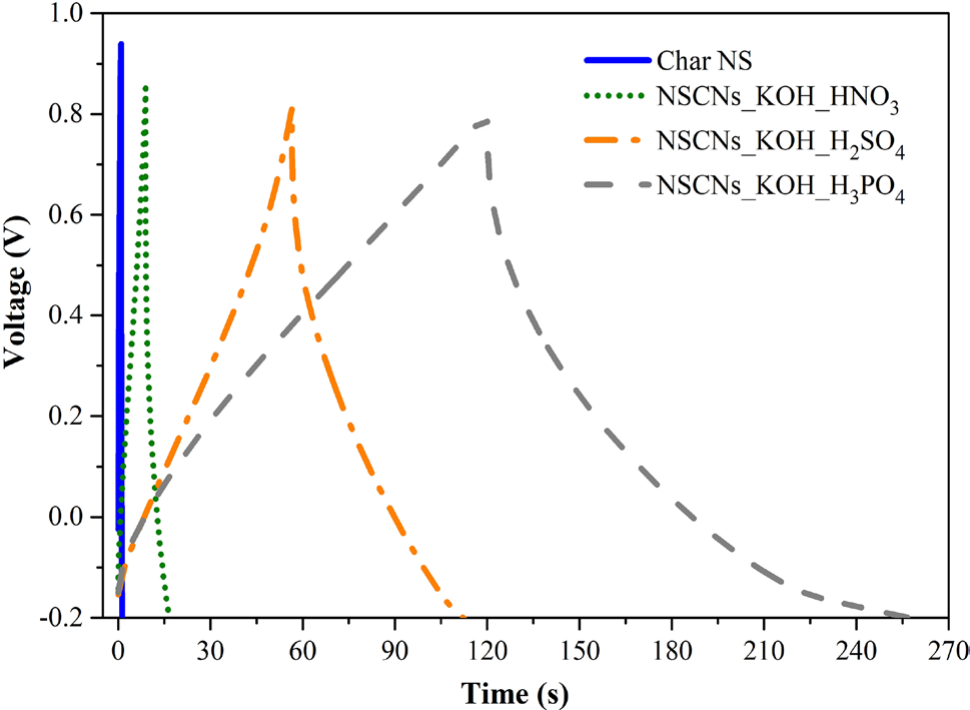
Galvanostatic charge/discharge of samples at a current density of 0.05 A g^−1^.

The galvanostatic charge/discharge (GCD) curves (Fig. 12) of the samples at a current density of 0.05 A g^−1^ in the voltage range of − 0.2 to 0.8 V show typical triangular shapes. The GCD curves are imperfectly symmetrical; they are slightly distorted due to the pseudocapacitive behaviour^55^, which is consistent with the CV graphs. The GCD curves show that NSCNs exfoliated by H_3_PO_4_ have the longest charge and discharge cycles, which implies the best electrochemical performance of the samples.

**Figure 13:**
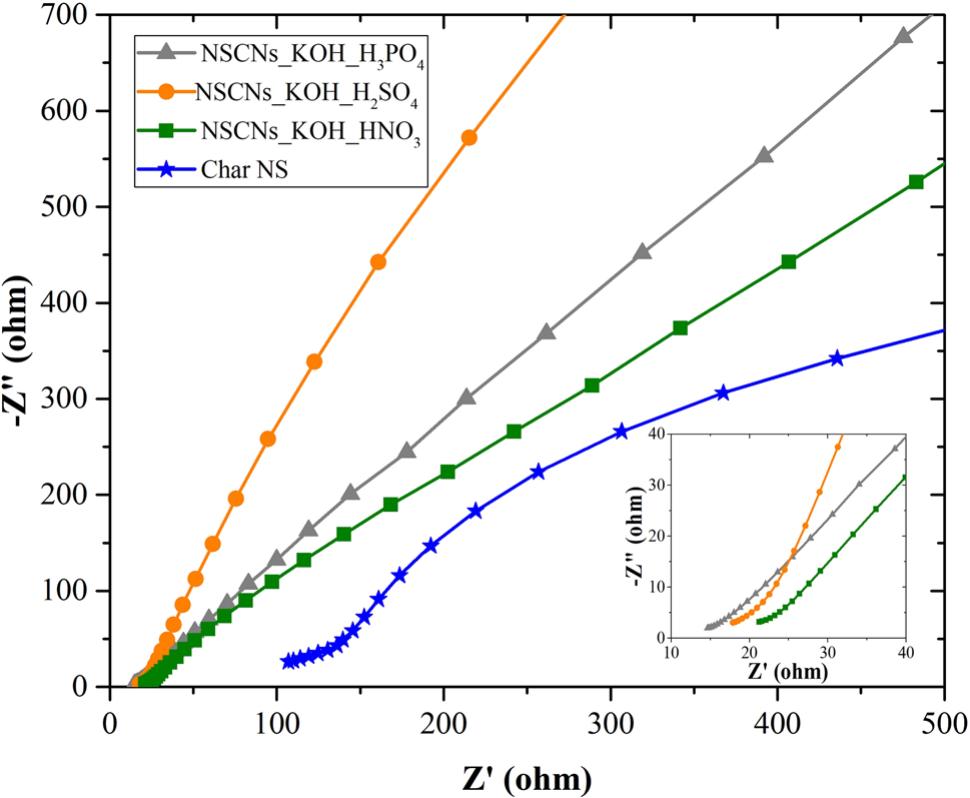
Nyquist plots of samples at an AC amplitude of 5 mV.

The electrochemical impedance spectroscopy (EIS) of NSCNs samples is presented in Fig. 13 with the Nyquist plots of each NSCNs electrode material in the frequency range between 0.1 Hz and 100 kHz. The slope of the straight line is close to 45° in the middle-high frequency and is assumed to the diffusion of the electrolyte ions in the electrode pores. The steep linear curve in the low frequency region of NSCNs is sharp, which is representative of diffusion-limited charge transfer characteristic close to ideal capacitance performance^56–59^. The equivalent series resistances (ESR) of NSCNs can be determined from the offsets on the x-axis in the high-frequency region. The ESRs of NSCNs exfoliated by H_3_PO_4_, H_2_SO_4_, HNO_3_ and char NS were approximately 14.6, 17.8 and 21.3 Ω, respectively, lower than that of char NS (107.0 Ω). The results confirmed the best electrochemical performance of NSCNs exfoliated by H_3_PO_4_. This observation is in agreement with the linear correlation between the peak current and square root of the scan rate in Fig. 11.

**Figure 14:**
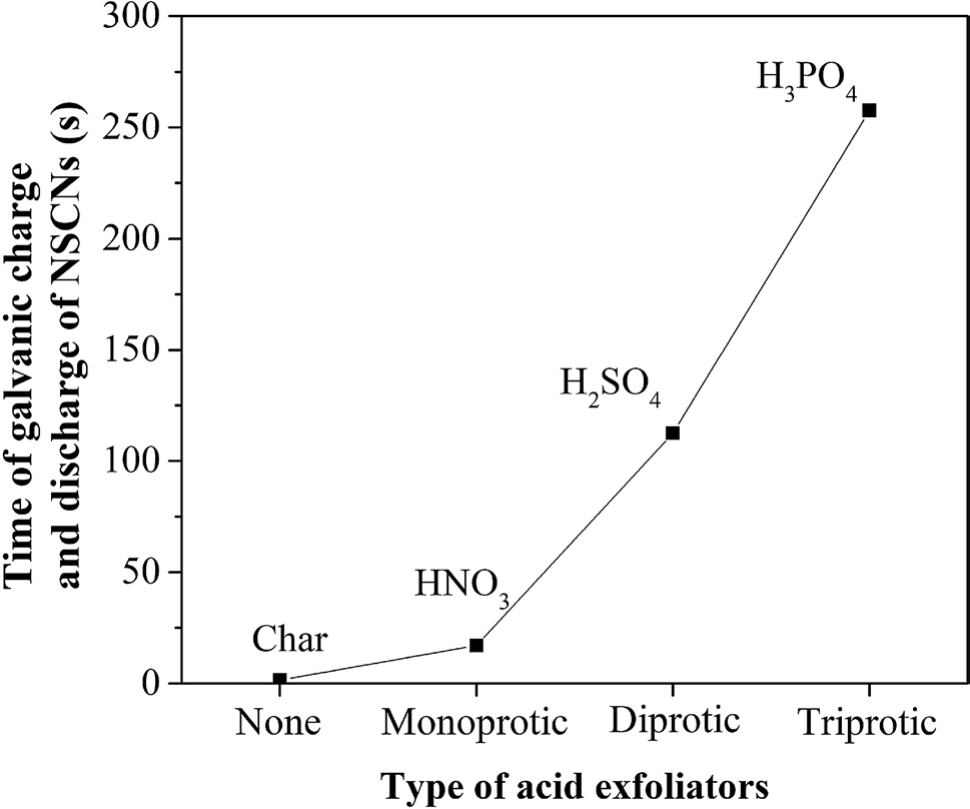
The time of galvanic charge and discharge of NSCNs with different types of acid exfoliators electrode.

The increase of dissociable protons (polyprotic acid) affects the longer charge and discharge cycle time, which results has the same effect as the specific surface area. The type of acid exfoliator affected the charge and discharge cycles. Cycle time for triprotic (H_3_PO_4_), diprotic (H_2_SO_4_) and monoprotic (HNO_3_) exfoliators was 260, 112 and 17 s, respectively (Fig. 14).

## Conclusions

Through the preparation method proposed, carbon nanosheets were successfully synthesized using nettle stems by different acid exfoliators (HNO_3_, H_2_SO_4_ or H_3_PO_4_). The morphological characteristics, chemical composition, specific surface area and micropore volume were intensively investigated. The results obtained in this work show that carbon nanosheets could be potential materials for capacitor application use as electrode material in energy storage devices. The results can be summarized as follows:

- The proposed method yields a high amount (> 80 wt.%) of carbon and clear formation of carbon nanosheet structures.
- K_3_PO_4_ is obtained from the reaction between the interlayered KOH residue and exfoliation acids (H_3_PO_4_), most likely due to its most negative ΔfG.
- Phosphoric acid exfoliates nettle stem carbon nanosheets (NSCNs) better than sulfuric acid, resulting in higher carbon content yield and higher specific surface area.
- The micropore volume of NSCNs exfoliated by phosphoric acid is higher than that of sulfuric or nitric acid; as a triprotic acid, it can react more intensively than other acids.
- Pore diameters in the exfoliated NSCNs were found to be less than 3 nm, which belongs to the micropore range (according to the IUPAC classification type I isotherm).
- NSCNs show higher specific capacitance compared to char samples (nettle stems without activation and exfoliation). The specific capacitance of NSCNs activated with KOH and exfoliated by H_3_PO_4_ reached 27.3 F g^−1^ at a scan rate of 5 mV s^−1^.
- Energy density (0.06–0.95 Wh kg^−1^) and power density (20.9–26.7 W kg^−1^) of NSCNs (at a current density of 0.05 A g^−1^) in this work confirmed to be supercapacitor materials.

## Methods

### Organic composition

The organic composition of the nettle stem, collected from Miskolc (Hungary), was investigated using chemical analysis at the commercial laboratory Mezőlabor Szolgáltató és Kereskedelmi Kft, Hungary. The acid detergent fibre content (ADF), acid detergent lignin content (ADL) and neutral detergent fibre content (NDF) were determined. The cellulose content was calculated by subtracting ADF from ADL. Similarly, hemicellulose content was calculated by subtracting NDF from ADF and lignin was calculated using ADL^35^.

### Synthesis of carbon nanosheets from nettle stem

The synthesis of carbon nanostructured materials from nettle stem was carried out as explained below:

- Sample preparation: The stem was chopped, washed and dried at 80 °C for 24 h followed by cleaning with HCl [0.5 M] for another 24 h. This facilitates the removal of organic compounds and residual metallic oxides^23^. It was finally washed with distilled water and dried at 80 °C for 24 h.
- Pre-Carbonization: The chopped stem was pre-carbonized (char NS) in a stainless-steel tubular furnace at 450 °C for 2 h under argon environment.
- Activation: The nettle stem was milled with a mortar for 1 h before being adding to aqueous KOH [1 M solute] with a weight ratio of 1:1 [KOH: Pre-carbonized nettle]. The mixture was stirred for an hour and dried at 80 °C for 24 h.
- Carbonization: The activated sample thus obtained was carbonized under Ar atmosphere in a tubular furnace for about 2 h. The temperature for the process was maintained at 800 °C.
- Exfoliation: The process of exfoliation was carried out by three different acids; hence, three different samples were obtained. 10 vol% each of sulfuric acid^28^, phosphoric acid or nitric acid were used as exfoliators. The samples were stirred for 1 h and washed with distilled water several times until a neutral pH was obtained. The samples were then dried at 80 °C for 24 h before subjecting them to characterization tests.

### Characterization

The exfoliated samples were coated with gold and investigated by scanning electron microscopy (SEM, Zeiss EVO-MA 10). The nanosheet structure was investigated by transmission electron microscopy (TEM, FEI TECNAI G2 20 X-TWIN). The chemical composition was analysed by energy-dispersive X-Ray spectrometry (EDS, EDAX Genesis). The specific surface area of the CNs was examined by Brunauer–Emmett–Teller method (BET, Micrometrics TriStar 3000) and for crystal structure X-ray Diffraction was used (XRD, Bruker D8 Advance diffractometer using Cu Kα radiation 40KV, 40 mA, in parallel beam geometry obtained with Gobel mirror, equipped with Vantec-1 position sensitive detector (1° window opening)). Patterns were recorded at 0.007° speed 2θ/29 s and within 2°–100° angular range of 2θ.

### Characterization of electrochemical properties

For characterizing electrochemical properties, synthesized nettle sample, carbon black and PTFE poly-tetrafluoroethylene were weighed in a mass ratio of 90:5:5 and mixed in mortar. The electrode material was characterized by a three-electrode system of NSCNs, Pt and Ag/AgCl where the latter two are used as the counter electrode and reference electrode respectively. 1 M sodium sulphate (Na_2_SO_4_) aqueous solution was used as electrolyte. The electrochemical properties of the electrode material and cell were characterized by cyclic voltammetry (CV), galvanostatic charge–discharge (GCD), and electrochemical impedance spectroscopy (EIS). The specific capacitance of a symmetric supercapacitor (Cs) is calculated by the following Eq. (1)^60^.

1 $$C_{s} = \frac{I \times \Delta t}{{\Delta V \times m}}$$

where *Cs, I, Δt, ΔV* and *m* are the specific capacitance (F g^−1^), discharge current (A), discharge time (s), voltage change after a full charge or discharge (V) and mass of active material on electrode (g), respectively. Energy density (E, Wh kg^−1^) (2) and power density (P, W kg^−1^) (3) of the electrode were calculated based on the following equations:

2 $${\text{E}} = \frac{{C_{s } \times \Delta V^{2} \times 1000}}{2 \times 3600}$$

3 $$P = \frac{E}{\Delta t} \times 3600$$
